# Supplementary material for: Effect of sanitation improvements on soil-transmitted helminth eggs in courtyard soil from rural Bangladesh: Evidence from a cluster-randomized controlled trial
Source: PLoS Negl Trop Dis. 2021 Jul 28;15(7):e0008815. doi: 10.1371/journal.pntd.0008815 (PMC8351931; doi:10.1371/journal.pntd.0008815)
Supplement: S1 Appendix — Table A: Unadjusted and adjusted prevalence ratio and prevalence difference for sanitation intervention vs. control arm for all soil-transmitted helminths and larvated soil-transmitted helminths. Table B: Unadjusted and adjusted egg count reduction for sanitation intervention vs. control arm for all soil-transmitted helminths and larvated soil-transmitted helminths. Table C: Prevalence of all soil-transmitted helminths in control arm, by subgroup. Table D: Concentration of all soil-transmitted helminths in control arm, by subgroup. Table E: Unadjusted prevalence ratio and prevalence difference for sanitation intervention vs. control arm for soil-transmitted helminth eggs, by subgroups. Table F: Unadjusted prevalence ratio and prevalence difference for sanitation intervention vs. control arm for larvated soil-transmitted helminth eggs, by subgroups. Table G: Unadjusted egg count reduction for sanitation intervention vs. control arm for soil-transmitted helminth eggs, by subgroups. Table H: Unadjusted egg count reduction for sanitation intervention vs. control arm for larvated soil-transmitted helminth eggs, by subgroups. Fig A: Prevalence difference for total and larvated A. lumbricoides eggs, T. trichiura eggs, any soil-transmitted helminth eggs, and multiple species of soil-transmitted helminth eggs, by subgroup. (DOCX) [file pntd.0008815.s001.docx]

S1 Appendix

**Effect of sanitation improvements on soil-transmitted helminth eggs in courtyard soil from rural Bangladesh: Evidence from a cluster-randomized controlled trial**

Table A: Unadjusted and adjusted prevalence ratio and prevalence difference for sanitation intervention *vs.* control arm for all soil-transmitted helminths and larvated soil-transmitted helminths

|  | | **Control** | | **Sanitation** | | **Prevalence ratio** | | **Prevalence difference** | |
| --- | --- | --- | --- | --- | --- | --- | --- | --- | --- |
| **Model** | **STH** | **N** | **mean** | **N** | **mean** | **PR (95% CI)** | **p-value** | **PD (95% CI)** | **p-value** |
| **All eggs** | | | | | | | | | |
| Unadjusted | *A. lumbricoides* | 914 | 0.63 | 491 | 0.61 | 0.97 (0.88, 1.06) | 0.51 | -0.02 (-0.08, 0.04) | 0.51 |
| Unadjusted | *T. trichiura* | 914 | 0.56 | 491 | 0.57 | 1.03 (0.92, 1.15) | 0.61 | 0.02 (-0.05, 0.08) | 0.61 |
| Unadjusted | Any STH | 914 | 0.76 | 491 | 0.74 | 0.98 (0.91, 1.05) | 0.59 | -0.02 (-0.07, 0.04) | 0.58 |
| Unadjusted | Multiple STH | 914 | 0.43 | 491 | 0.44 | 1.03 (0.90, 1.19) | 0.68 | 0.01 (-0.05, 0.07) | 0.68 |
| **Larvated eggs** | | | | | | | | | |
| Unadjusted | *A. lumbricoides* | 914 | 0.55 | 491 | 0.53 | 0.96 (0.86, 1.07) | 0.48 | -0.02 (-0.08, 0.04) | 0.48 |
| Unadjusted | *T. trichiura* | 914 | 0.46 | 491 | 0.48 | 1.03 (0.90, 1.19) | 0.65 | 0.02 (-0.05, 0.08) | 0.66 |
| Unadjusted | Any STH | 914 | 0.67 | 491 | 0.66 | 0.98 (0.90, 1.07) | 0.68 | -0.01 (-0.07, 0.05) | 0.68 |
| Unadjusted | Multiple STH | 914 | 0.34 | 491 | 0.34 | 1.02 (0.85, 1.22) | 0.81 | 0.01 (-0.05, 0.07) | 0.81 |
| **All eggs** | | | | | | | | | |
| Adjusted | *A. lumbricoides* | 914 | 0.63 | 491 | 0.61 | 0.96 (0.89, 1.05) | 0.37 | -0.02 (-0.07, 0.03) | 0.37 |
| Adjusted | *T. trichiura* | 914 | 0.56 | 491 | 0.57 | 1.02 (0.92, 1.13) | 0.70 | 0.01 (-0.05, 0.07) | 0.70 |
| Adjusted | Any STH | 914 | 0.76 | 491 | 0.74 | 0.98 (0.92, 1.04) | 0.49 | -0.02 (-0.06, 0.03) | 0.49 |
| Adjusted | Multiple STH | 914 | 0.43 | 491 | 0.44 | 1.01 (0.89, 1.15) | 0.89 | 0.00 (-0.05, 0.06) | 0.89 |
| **Larvated eggs** | | | | | | | | | |
| Adjusted | *A. lumbricoides* | 914 | 0.55 | 491 | 0.53 | 0.95 (0.86, 1.05) | 0.30 | -0.03 (-0.08, 0.02) | 0.30 |
| Adjusted | *T. trichiura* | 914 | 0.46 | 491 | 0.48 | 1.02 (0.90, 1.16) | 0.77 | 0.01 (-0.05, 0.07) | 0.77 |
| Adjusted | Any STH | 914 | 0.67 | 491 | 0.66 | 0.98 (0.90, 1.06) | 0.56 | -0.02 (-0.07, 0.04) | 0.56 |
| Adjusted | Multiple STH | 914 | 0.34 | 491 | 0.34 | 0.99 (0.84, 1.16) | 0.88 | -0.00 (-0.06, 0.05) | 0.88 |

STH = soil-transmitted helminth; 95% CI = 95% confidence interval

# Table B: Unadjusted and adjusted egg count reduction for sanitation intervention *vs.* control arm for all soil-transmitted helminths and larvated soil-transmitted helminths

|  | | **Control** | | | **Sanitation** | | | **Arithmetic** | | **Geometric** | |
| --- | --- | --- | --- | --- | --- | --- | --- | --- | --- | --- | --- |
| **Model** | **STH** | **N** | **ari**  **mean** | **geo mean** | **N** | **ari mean** | **geo mean** | **ECR (95% CI)** | **p-value** | **ECR (95% CI)** | **p-value** |
| **All eggs** | | | | | | | | | | | |
| Unadjusted | *A. lumbricoides* | 914 | 2.33 | 0.08 | 491 | 2.26 | 0.07 | -0.03 (-0.38, 0.32) | 0.87 | -0.01 (-0.14, 0.13) | 0.93 |
| Unadjusted | *T. trichiura* | 914 | 1.62 | -0.08 | 491 | 1.98 | 0.03 | 0.23 (-0.16, 0.61) | 0.25 | 0.12 (-0.02, 0.25) | 0.09 |
| Unadjusted | Any STH | 914 | 3.96 | 0.45 | 491 | 4.24 | 0.53 | 0.07 (-0.22, 0.37) | 0.62 | 0.08 (-0.10, 0.26) | 0.36 |
| **Larvated eggs** | | | | | | | | | | | |
| Unadjusted | *A. lumbricoides* | 914 | 1.96 | 0.00 | 491 | 1.83 | -0.02 | -0.07 (-0.42, 0.29) | 0.70 | -0.03 (-0.15, 0.10) | 0.68 |
| Unadjusted | *T. trichiura* | 914 | 1.24 | -0.19 | 491 | 1.49 | -0.08 | 0.21 (-0.19, 0.61) | 0.31 | 0.11 (-0.01, 0.24) | 0.07 |
| Unadjusted | Any STH | 914 | 3.20 | 0.30 | 491 | 3.32 | 0.35 | 0.04 (-0.27, 0.34) | 0.81 | 0.05 (-0.11, 0.21) | 0.54 |
| **All eggs** | | | | | | | | | | | |
| Adjusted | *A. lumbricoides* | 914 | 2.33 | 0.08 | 491 | 2.26 | 0.07 | -0.06 (-0.39, 0.26) | 0.69 | -0.05 (-0.16, 0.06) | 0.39 |
| Adjusted | *T. trichiura* | 914 | 1.62 | -0.08 | 491 | 1.98 | 0.03 | 0.16 (-0.18, 0.51) | 0.36 | 0.09 (-0.03, 0.20) | 0.14 |
| Adjusted | Any STH | 914 | 3.96 | 0.45 | 491 | 4.24 | 0.53 | 0.02 (-0.24, 0.28) | 0.87 | 0.03 (-0.11, 0.17) | 0.69 |
| **Larvated eggs** | | | | | | | | | | | |
| Adjusted | *A. lumbricoides* | 914 | 1.96 | 0.00 | 491 | 1.83 | -0.02 | -0.10 (-0.43, 0.23) | 0.56 | -0.06 (-0.16, 0.04) | 0.21 |
| Adjusted | *T. trichiura* | 914 | 1.24 | -0.19 | 491 | 1.49 | -0.08 | 0.14 (-0.22, 0.50) | 0.43 | 0.08 (-0.02, 0.19) | 0.12 |
| Adjusted | Any STH | 914 | 3.20 | 0.30 | 491 | 3.32 | 0.35 | -0.01 (-0.28, 0.25) | 0.92 | 0.00 (-0.13, 0.13) | 1.00 |

STH = soil-transmitted helminth; 95% CI = 95% confidence interval

Table C: Prevalence of all soil-transmitted helminths in control arm, by subgroup

|  | **Season** | | **Individuals per compound** | | **Compound-level deworming** | | **Cluster-level deworming** | |
| --- | --- | --- | --- | --- | --- | --- | --- | --- |
|  | **Dry (n=686)** | **Wet  (n=228)** | **<10  (n=437)** | **10+  (n=477)** | **<2/3  (n=396)** | **2/3+  (n=516)** | **<2/3  (n=339)** | **2/3+  (n=483)** |
| **STH** | **mean (95% CI)** | **mean (95% CI)** | **mean (95% CI)** | **mean (95% CI)** | **mean (95% CI)** | **mean (95% CI)** | **mean (95% CI)** | **mean (95% CI)** |
| **Total STH** | | | | | | | | |
| *A. lumbricoides* | 0.62 (0.57, 0.66) | 0.67 (0.59, 0.74) | 0.63 (0.58, 0.68) | 0.63 (0.58, 0.69) | 0.62 (0.56, 0.68) | 0.64 (0.59, 0.69) | 0.62 (0.56, 0.68) | 0.65 (0.60, 0.70) |
| *T. trichiura* | 0.61 (0.57, 0.65) | 0.40 (0.32, 0.49) | 0.54 (0.48, 0.60) | 0.57 (0.52, 0.62) | 0.55 (0.49, 0.61) | 0.56 (0.51, 0.62) | 0.57 (0.52, 0.63) | 0.56 (0.51, 0.61) |
| Any STH | 0.76 (0.72, 0.80) | 0.76 (0.69, 0.83) | 0.76 (0.71, 0.80) | 0.76 (0.72, 0.80) | 0.75 (0.70, 0.81) | 0.76 (0.72, 0.81) | 0.75 (0.70, 0.80) | 0.77 (0.73, 0.82) |
| Multiple STH | 0.47 (0.42, 0.52) | 0.31 (0.23, 0.39) | 0.41 (0.36, 0.46) | 0.45 (0.39, 0.50) | 0.41 (0.35, 0.48) | 0.44 (0.39, 0.50) | 0.44 (0.38, 0.50) | 0.43 (0.38, 0.48) |
| **Larvated STH** | | | | | | | | |
| *A. lumbricoides* | 0.54 (0.49, 0.58) | 0.58 (0.50, 0.67) | 0.55 (0.50, 0.60) | 0.55 (0.50, 0.60) | 0.54 (0.48, 0.60) | 0.55 (0.50, 0.61) | 0.53 (0.47, 0.59) | 0.57 (0.51, 0.62) |
| *T. trichiura* | 0.49 (0.45, 0.54) | 0.37 (0.28, 0.45) | 0.47 (0.41, 0.52) | 0.46 (0.40, 0.51) | 0.44 (0.38, 0.51) | 0.47 (0.42, 0.53) | 0.48 (0.42, 0.54) | 0.45 (0.40, 0.51) |
| Any STH | 0.67 (0.62, 0.71) | 0.68 (0.60, 0.77) | 0.68 (0.63, 0.74) | 0.66 (0.62, 0.71) | 0.67 (0.61, 0.73) | 0.68 (0.63, 0.73) | 0.66 (0.60, 0.72) | 0.69 (0.64, 0.73) |
| Multiple STH | 0.36 (0.32, 0.41) | 0.27 (0.19, 0.34) | 0.34 (0.29, 0.38) | 0.34 (0.29, 0.39) | 0.32 (0.27, 0.38) | 0.35 (0.30, 0.41) | 0.35 (0.30, 0.40) | 0.33 (0.28, 0.38) |

STH = soil-transmitted helminth; 95% CI = 95% confidence interval

# Table D: Concentration of all soil-transmitted helminths in control arm, by subgroup

|  | **Season** | | **Individuals per compound** | | **Compound-level deworming** | | **Cluster-level deworming** | |
| --- | --- | --- | --- | --- | --- | --- | --- | --- |
|  | **Dry  (n=686)** | **Wet  (n=228)** | **<10  (n=437)** | **10+  (n=477)** | **<2/3  (n=396)** | **2/3+  (n=516)** | **<2/3  (n=339)** | **2/3+  (n=483)** |
| **STH** | **mean (95% CI)** | **mean (95% CI)** | **mean (95% CI)** | **mean (95% CI)** | **mean (95% CI)** | **mean (95% CI)** | **mean (95% CI)** | **mean (95% CI)** |
| **Arithmetic** | | | | | | | | |
| **Total STH** | | | | | | | | |
| *A. lumbricoides* | 2.71 (2.06, 3.36) | 1.20 (0.87, 1.53) | 1.81 (1.41, 2.22) | 2.81 (2.00, 3.61) | 2.01 (1.47, 2.55) | 2.58 (1.80, 3.37) | 2.49 (1.51, 3.47) | 2.33 (1.75, 2.91) |
| *T. trichiura* | 1.98 (1.51, 2.44) | 0.57 (0.37, 0.77) | 1.62 (1.01, 2.23) | 1.63 (1.28, 1.98) | 1.32 (1.00, 1.64) | 1.86 (1.27, 2.46) | 1.42 (1.12, 1.71) | 1.84 (1.26, 2.42) |
| Any STH | 4.68 (3.73, 5.64) | 1.77 (1.30, 2.23) | 3.44 (2.49, 4.38) | 4.43 (3.42, 5.45) | 3.33 (2.59, 4.07) | 4.45 (3.24, 5.65) | 3.90 (2.74, 5.07) | 4.17 (3.17, 5.17) |
| **Larvated STH** | | | | | | | | |
| *A. lumbricoides* | 2.28 (1.69, 2.86) | 1.03 (0.73, 1.32) | 1.54 (1.19, 1.90) | 2.35 (1.62, 3.08) | 1.70 (1.22, 2.18) | 2.17 (1.46, 2.88) | 2.16 (1.24, 3.08) | 1.92 (1.43, 2.42) |
| *T. trichiura* | 1.50 (1.09, 1.91) | 0.46 (0.30, 0.61) | 1.28 (0.72, 1.83) | 1.20 (0.92, 1.48) | 0.95 (0.71, 1.19) | 1.46 (0.93, 1.99) | 1.05 (0.81, 1.29) | 1.42 (0.91, 1.94) |
| Any STH | 3.77 (2.92, 4.63) | 1.48 (1.09, 1.87) | 2.82 (1.98, 3.66) | 3.55 (2.65, 4.45) | 2.65 (2.02, 3.28) | 3.63 (2.55, 4.71) | 3.21 (2.14, 4.28) | 3.35 (2.47, 4.22) |
| **Geometric** | | | | | | | | |
| **Total STH** | | | | | | | | |
| *A. lumbricoides* | 0.19 (0.07, 0.31) | -0.27 (-0.47, -0.07) | -0.02 (-0.15, 0.10) | 0.17 (0.04, 0.30) | 0.07 (-0.08, 0.21) | 0.09 (-0.07, 0.24) | 0.06 (-0.08, 0.20) | 0.10 (-0.03, 0.24) |
| *T. trichiura* | 0.06 (-0.04, 0.17) | -0.49 (-0.61, -0.37) | -0.14 (-0.25, -0.02) | -0.02 (-0.13, 0.09) | -0.10 (-0.23, 0.03) | -0.05 (-0.19, 0.08) | -0.05 (-0.16, 0.06) | -0.05 (-0.16, 0.06) |
| Any STH | 0.63 (0.48, 0.77) | -0.06 (-0.30, 0.18) | 0.36 (0.21, 0.52) | 0.54 (0.38, 0.70) | 0.45 (0.27, 0.63) | 0.46 (0.27, 0.65) | 0.46 (0.30, 0.62) | 0.48 (0.32, 0.64) |
| **Larvated STH** | | | | | | | | |
| *A. lumbricoides* | 0.09 (-0.02, 0.20) | -0.25 (-0.42, -0.09) | -0.06 (-0.17, 0.05) | 0.06 (-0.06, 0.19) | -0.02 (-0.14, 0.11) | 0.02 (-0.12, 0.16) | -0.01 (-0.13, 0.12) | 0.02 (-0.10, 0.14) |
| *T. trichiura* | -0.08 (-0.18, 0.01) | -0.51 (-0.62, -0.40) | -0.23 (-0.34, -0.13) | -0.15 (-0.25, -0.06) | -0.22 (-0.32, -0.12) | -0.16 (-0.28, -0.04) | -0.19 (-0.29, -0.09) | -0.16 (-0.26, -0.06) |
| Any STH | 0.43 (0.29, 0.57) | -0.08 (-0.27, 0.12) | 0.26 (0.12, 0.39) | 0.35 (0.20, 0.49) | 0.28 (0.13, 0.44) | 0.32 (0.15, 0.49) | 0.30 (0.15, 0.45) | 0.33 (0.19, 0.48) |

STH = soil-transmitted helminth; 95% CI = 95% confidence interval

# Table E: Unadjusted prevalence ratio and prevalence difference for sanitation intervention vs. control arm for soil-transmitted helminth eggs, by subgroups

|  | **Control** | | **Sanitation** | | **Prevalence ratio** | | **Prevalence difference** | | |
| --- | --- | --- | --- | --- | --- | --- | --- | --- | --- |
| **All eggs** | **N** | **mean** | **N** | **mean** | **PR (95% CI)** | **p-value** | | **PD (95% CI)** | **p-value** |
| **Total** | | | | | | | | | |
| *A. lumbricoides* | 914 | 0.63 | 491 | 0.61 | 0.97 (0.88, 1.06) | 0.51 | | -0.02 (-0.08, 0.04) | 0.51 |
| *T. trichiura* | 914 | 0.56 | 491 | 0.57 | 1.03 (0.92, 1.15) | 0.61 | | 0.02 (-0.05, 0.08) | 0.61 |
| Any STH | 914 | 0.76 | 491 | 0.74 | 0.98 (0.91, 1.05) | 0.59 | | -0.02 (-0.07, 0.04) | 0.58 |
| Multiple STH | 914 | 0.43 | 491 | 0.44 | 1.03 (0.90, 1.19) | 0.68 | | 0.01 (-0.05, 0.07) | 0.68 |
| **Season** | | | | | | | | | |
| **Dry** | | | | | | | | | |
| *A. lumbricoides* | 686 | 0.62 | 371 | 0.60 | 0.96 (0.83, 1.12) | 0.63 | | -0.02 (-0.11, 0.07) | 0.63 |
| *T. trichiura* | 686 | 0.61 | 371 | 0.63 | 1.03 (0.89, 1.20) | 0.68 | | 0.02 (-0.07, 0.12) | 0.68 |
| Any STH | 686 | 0.76 | 371 | 0.73 | 0.97 (0.86, 1.09) | 0.61 | | -0.02 (-0.11, 0.07) | 0.61 |
| Multiple STH | 686 | 0.47 | 371 | 0.49 | 1.04 (0.86, 1.26) | 0.66 | | 0.02 (-0.07, 0.11) | 0.67 |
| **Wet** | | | | | | | | | |
| *A. lumbricoides* | 228 | 0.67 | 120 | 0.66 | 0.99 (0.79, 1.24) | 0.91 | | -0.01 (-0.16, 0.14) | 0.91 |
| *T. trichiura* | 228 | 0.40 | 120 | 0.40 | 0.99 (0.69, 1.42) | 0.97 | | -0.00 (-0.15, 0.14) | 0.97 |
| Any STH | 228 | 0.76 | 120 | 0.77 | 1.01 (0.83, 1.24) | 0.91 | | 0.01 (-0.15, 0.16) | 0.91 |
| Multiple STH | 228 | 0.31 | 120 | 0.29 | 0.94 (0.62, 1.43) | 0.77 | | -0.02 (-0.14, 0.11) | 0.76 |
| **People per compound** | | | | | | | | | |
| **<10** | | | | | | | | | |
| *A. lumbricoides* | 437 | 0.63 | 241 | 0.61 | 0.97 (0.84, 1.12) | 0.70 | | -0.02 (-0.11, 0.07) | 0.70 |
| *T. trichiura* | 437 | 0.54 | 241 | 0.54 | 1.01 (0.87, 1.18) | 0.91 | | 0.00 (-0.08, 0.09) | 0.91 |
| Any STH | 437 | 0.76 | 241 | 0.74 | 0.98 (0.87, 1.10) | 0.72 | | -0.02 (-0.10, 0.07) | 0.72 |
| Multiple STH | 437 | 0.41 | 241 | 0.41 | 1.01 (0.83, 1.23) | 0.91 | | 0.00 (-0.08, 0.09) | 0.91 |
| **10+** | | | | | | | | | |
| *A. lumbricoides* | 477 | 0.63 | 250 | 0.61 | 0.97 (0.83, 1.13) | 0.66 | | -0.02 (-0.12, 0.07) | 0.66 |
| *T. trichiura* | 477 | 0.57 | 250 | 0.60 | 1.05 (0.88, 1.25) | 0.59 | | 0.03 (-0.08, 0.13) | 0.60 |
| Any STH | 477 | 0.76 | 250 | 0.74 | 0.98 (0.88, 1.10) | 0.77 | | -0.01 (-0.10, 0.07) | 0.77 |
| Multiple STH | 477 | 0.45 | 250 | 0.47 | 1.04 (0.83, 1.31) | 0.70 | | 0.02 (-0.08, 0.12) | 0.70 |
| **Proportion of children in compound who were dewormed** | | | | | | | | | |
| **<2/3** | | | | | | | | | |
| *A. lumbricoides* | 396 | 0.62 | 221 | 0.65 | 1.06 (0.89, 1.26) | 0.50 | | 0.04 (-0.07, 0.15) | 0.50 |
| *T. trichiura* | 396 | 0.55 | 221 | 0.61 | 1.11 (0.90, 1.36) | 0.33 | | 0.06 (-0.06, 0.18) | 0.33 |
| Any STH | 396 | 0.75 | 221 | 0.75 | 0.99 (0.86, 1.13) | 0.89 | | -0.01 (-0.11, 0.09) | 0.89 |
| Multiple STH | 396 | 0.41 | 221 | 0.51 | 1.24 (0.95, 1.61) | 0.12 | | 0.10 (-0.02, 0.22) | 0.12 |
| **2/3+** | | | | | | | | | |
| *A. lumbricoides* | 516 | 0.64 | 270 | 0.58 | 0.91 (0.79, 1.04) | 0.16 | | -0.06 (-0.14, 0.02) | 0.16 |
| *T. trichiura* | 516 | 0.56 | 270 | 0.54 | 0.97 (0.78, 1.19) | 0.75 | | -0.02 (-0.14, 0.10) | 0.75 |
| Any STH | 516 | 0.76 | 270 | 0.74 | 0.97 (0.86, 1.09) | 0.57 | | -0.02 (-0.11, 0.06) | 0.57 |
| Multiple STH | 516 | 0.44 | 270 | 0.39 | 0.87 (0.69, 1.11) | 0.26 | | -0.06 (-0.15, 0.04) | 0.25 |
| **Proportion of children in cluster who were dewormed** | | | | | | | | | |
| **<2/3** | | | | | | | | | |
| *A. lumbricoides* | 339 | 0.62 | 214 | 0.65 | 1.05 (0.89, 1.23) | 0.56 | | 0.03 (-0.07, 0.13) | 0.56 |
| *T. trichiura* | 339 | 0.57 | 214 | 0.57 | 1.00 (0.84, 1.20) | 0.97 | | 0.00 (-0.10, 0.10) | 0.97 |
| Any STH | 339 | 0.75 | 214 | 0.76 | 1.01 (0.90, 1.13) | 0.85 | | 0.01 (-0.08, 0.09) | 0.85 |
| Multiple STH | 339 | 0.44 | 214 | 0.47 | 1.06 (0.85, 1.33) | 0.60 | | 0.03 (-0.08, 0.13) | 0.60 |
| **2/3+** | | | | | | | | | |
| *A. lumbricoides* | 483 | 0.65 | 263 | 0.57 | 0.89 (0.77, 1.02) | 0.10 | | -0.07 (-0.16, 0.01) | 0.09 |
| *T. trichiura* | 483 | 0.56 | 263 | 0.57 | 1.03 (0.89, 1.20) | 0.70 | | 0.02 (-0.07, 0.10) | 0.70 |
| Any STH | 483 | 0.77 | 263 | 0.73 | 0.94 (0.84, 1.06) | 0.32 | | -0.04 (-0.13, 0.04) | 0.31 |
| Multiple STH | 483 | 0.43 | 263 | 0.42 | 0.97 (0.81, 1.16) | 0.73 | | -0.01 (-0.09, 0.06) | 0.73 |

STH = soil-transmitted helminth; 95% CI = 95% confidence interval

# Table F: Unadjusted prevalence ratio and prevalence difference for sanitation intervention vs. control arm for larvated soil-transmitted helminth eggs, by subgroups

|  | **Control** | | **Sanitation** | | **Prevalence ratio** | | **Prevalence difference** | |
| --- | --- | --- | --- | --- | --- | --- | --- | --- |
| **Larvated eggs** | **N** | **mean** | **N** | **mean** | **PR (95% CI)** | **p-value** | **PD (95% CI)** | **p-value** |
| **Total** | | | | | | | | |
| *A. lumbricoides* | 914 | 0.55 | 491 | 0.53 | 0.96 (0.86, 1.07) | 0.48 | -0.02 (-0.08, 0.04) | 0.48 |
| *T. trichiura* | 914 | 0.46 | 491 | 0.48 | 1.03 (0.90, 1.19) | 0.65 | 0.02 (-0.05, 0.08) | 0.66 |
| Any STH | 914 | 0.67 | 491 | 0.66 | 0.98 (0.90, 1.07) | 0.68 | -0.01 (-0.07, 0.05) | 0.68 |
| Multiple STH | 914 | 0.34 | 491 | 0.34 | 1.02 (0.85, 1.22) | 0.81 | 0.01 (-0.05, 0.07) | 0.81 |
| **Season** | | | | | | | | |
| **Dry** | | | | | | | | |
| *A. lumbricoides* | 686 | 0.54 | 371 | 0.51 | 0.95 (0.80, 1.12) | 0.52 | -0.03 (-0.12, 0.06) | 0.52 |
| *T. trichiura* | 686 | 0.49 | 371 | 0.51 | 1.04 (0.87, 1.24) | 0.68 | 0.02 (-0.07, 0.11) | 0.68 |
| Any STH | 686 | 0.67 | 371 | 0.65 | 0.97 (0.85, 1.11) | 0.67 | -0.02 (-0.11, 0.07) | 0.67 |
| Multiple STH | 686 | 0.36 | 371 | 0.37 | 1.03 (0.80, 1.31) | 0.84 | 0.01 (-0.08, 0.10) | 0.84 |
| **Wet** | | | | | | | | |
| *A. lumbricoides* | 228 | 0.58 | 120 | 0.58 | 1.00 (0.77, 1.29) | 1.00 | -0.00 (-0.15, 0.15) | 1.00 |
| *T. trichiura* | 228 | 0.37 | 120 | 0.37 | 1.00 (0.67, 1.47) | 0.99 | -0.00 (-0.14, 0.14) | 0.99 |
| Any STH | 228 | 0.68 | 120 | 0.69 | 1.01 (0.80, 1.28) | 0.92 | 0.01 (-0.15, 0.17) | 0.92 |
| Multiple STH | 228 | 0.27 | 120 | 0.26 | 0.97 (0.62, 1.51) | 0.88 | -0.01 (-0.13, 0.11) | 0.88 |
| **People per compound** | | | | | | | | |
| **<10** | | | | | | | | |
| *A. lumbricoides* | 437 | 0.55 | 241 | 0.53 | 0.96 (0.82, 1.13) | 0.64 | -0.02 (-0.11, 0.07) | 0.63 |
| *T. trichiura* | 437 | 0.47 | 241 | 0.48 | 1.04 (0.86, 1.24) | 0.71 | 0.02 (-0.07, 0.10) | 0.71 |
| Any STH | 437 | 0.68 | 241 | 0.68 | 1.00 (0.88, 1.14) | 0.96 | 0.00 (-0.09, 0.09) | 0.96 |
| Multiple STH | 437 | 0.34 | 241 | 0.33 | 0.98 (0.78, 1.23) | 0.87 | -0.01 (-0.08, 0.07) | 0.87 |
| **10+** | | | | | | | | |
| *A. lumbricoides* | 477 | 0.55 | 250 | 0.52 | 0.96 (0.80, 1.15) | 0.64 | -0.02 (-0.12, 0.07) | 0.64 |
| *T. trichiura* | 477 | 0.46 | 250 | 0.47 | 1.03 (0.82, 1.30) | 0.81 | 0.01 (-0.09, 0.12) | 0.81 |
| Any STH | 477 | 0.66 | 250 | 0.64 | 0.96 (0.82, 1.12) | 0.57 | -0.03 (-0.13, 0.07) | 0.56 |
| Multiple STH | 477 | 0.34 | 250 | 0.36 | 1.06 (0.80, 1.40) | 0.68 | 0.02 (-0.08, 0.12) | 0.69 |
| **Proportion of children in compound who were dewormed** | | | | | | | | |
| **<2/3** | | | | | | | | |
| *A. lumbricoides* | 396 | 0.54 | 221 | 0.54 | 0.99 (0.81, 1.21) | 0.93 | -0.01 (-0.12, 0.10) | 0.93 |
| *T. trichiura* | 396 | 0.44 | 221 | 0.51 | 1.15 (0.90, 1.49) | 0.27 | 0.07 (-0.05, 0.19) | 0.27 |
| Any STH | 396 | 0.67 | 221 | 0.67 | 1.00 (0.85, 1.18) | 0.97 | 0.00 (-0.11, 0.11) | 0.97 |
| Multiple STH | 396 | 0.32 | 221 | 0.38 | 1.19 (0.85, 1.67) | 0.31 | 0.06 (-0.06, 0.18) | 0.31 |
| **2/3+** | | | | | | | | |
| *A. lumbricoides* | 516 | 0.55 | 270 | 0.52 | 0.93 (0.80, 1.09) | 0.39 | -0.04 (-0.12, 0.05) | 0.39 |
| *T. trichiura* | 516 | 0.47 | 270 | 0.45 | 0.94 (0.74, 1.20) | 0.64 | -0.03 (-0.14, 0.08) | 0.64 |
| Any STH | 516 | 0.68 | 270 | 0.65 | 0.96 (0.84, 1.09) | 0.54 | -0.03 (-0.11, 0.06) | 0.54 |
| Multiple STH | 516 | 0.35 | 270 | 0.31 | 0.90 (0.68, 1.19) | 0.45 | -0.04 (-0.13, 0.06) | 0.45 |
| **Proportion of children in cluster who were dewormed** | | | | | | | | |
| **<2/3** | | | | | | | | |
| *A. lumbricoides* | 339 | 0.53 | 214 | 0.54 | 1.02 (0.84, 1.23) | 0.85 | 0.01 (-0.09, 0.11) | 0.85 |
| *T. trichiura* | 339 | 0.48 | 214 | 0.49 | 1.02 (0.81, 1.28) | 0.87 | 0.01 (-0.10, 0.12) | 0.87 |
| Any STH | 339 | 0.66 | 214 | 0.68 | 1.03 (0.90, 1.20) | 0.64 | 0.02 (-0.07, 0.12) | 0.64 |
| Multiple STH | 339 | 0.35 | 214 | 0.35 | 0.99 (0.74, 1.32) | 0.94 | -0.00 (-0.11, 0.10) | 0.94 |
| **2/3+** | | | | | | | | |
| *A. lumbricoides* | 483 | 0.57 | 263 | 0.52 | 0.91 (0.78, 1.07) | 0.27 | -0.05 (-0.13, 0.04) | 0.26 |
| *T. trichiura* | 483 | 0.45 | 263 | 0.46 | 1.03 (0.86, 1.23) | 0.78 | 0.01 (-0.07, 0.09) | 0.78 |
| Any STH | 483 | 0.69 | 263 | 0.64 | 0.93 (0.81, 1.07) | 0.30 | -0.05 (-0.14, 0.04) | 0.29 |
| Multiple STH | 483 | 0.33 | 263 | 0.34 | 1.03 (0.82, 1.29) | 0.80 | 0.01 (-0.07, 0.09) | 0.80 |

STH = soil-transmitted helminth; 95% CI = 95% confidence interval

# Table G: Unadjusted egg count reduction for sanitation intervention vs. control arm for soil-transmitted helminth eggs, by subgroups

|  | **Control** | | | **Sanitation** | | | **Arithmetic** | | **Geometric** | |
| --- | --- | --- | --- | --- | --- | --- | --- | --- | --- | --- |
| **All eggs** | **N** | **ari mean** | **geo mean** | **N** | **ari mean** | **geo mean** | **ECR (95% CI)** | **p-value** | **ECR (95% CI)** | **p-value** |
| **Total** | | | | | | | | | | |
| *A. lumbricoides* | 914 | 2.33 | 0.08 | 491 | 2.26 | 0.07 | -0.03 (-0.38, 0.32) | 0.87 | -0.01 (-0.14, 0.13) | 0.93 |
| *T. trichiura* | 914 | 1.62 | -0.08 | 491 | 1.98 | 0.03 | 0.23 (-0.16, 0.61) | 0.25 | 0.12 (-0.02, 0.25) | 0.09 |
| Any STH | 914 | 3.96 | 0.45 | 491 | 4.24 | 0.53 | 0.07 (-0.22, 0.37) | 0.62 | 0.08 (-0.10, 0.26) | 0.36 |
| **Season** | | | | | | | | | | |
| **Dry** | | | | | | | | | | |
| *A. lumbricoides* | 686 | 2.71 | 0.19 | 371 | 2.38 | 0.12 | -0.12 (-0.48, 0.24) | 0.51 | -0.07 (-0.28, 0.13) | 0.48 |
| *T. trichiura* | 686 | 1.98 | 0.06 | 371 | 2.12 | 0.15 | 0.07 (-0.27, 0.41) | 0.69 | 0.08 (-0.14, 0.31) | 0.46 |
| Any STH | 686 | 4.68 | 0.63 | 371 | 4.50 | 0.63 | -0.04 (-0.33, 0.24) | 0.78 | 0.00 (-0.27, 0.28) | 0.98 |
| **Wet** | | | | | | | | | | |
| *A. lumbricoides* | 228 | 1.20 | -0.27 | 120 | 1.88 | -0.07 | 0.53 (-0.33, 1.39) | 0.23 | 0.22 (-0.14, 0.57) | 0.23 |
| *T. trichiura* | 228 | 0.57 | -0.49 | 120 | 1.56 | -0.31 | 1.75 (-0.40, 3.90) | 0.11 | 0.19 (-0.06, 0.45) | 0.14 |
| Any STH | 228 | 1.77 | -0.06 | 120 | 3.44 | 0.23 | 0.96 (-0.29, 2.22) | 0.13 | 0.33 (-0.14, 0.81) | 0.16 |
| **People per compound** | | | | | | | | | | |
| **<10** | | | | | | | | | | |
| *A. lumbricoides* | 437 | 1.81 | -0.02 | 241 | 1.98 | 0.03 | 0.09 (-0.35, 0.54) | 0.68 | 0.06 (-0.15, 0.27) | 0.60 |
| *T. trichiura* | 437 | 1.62 | -0.14 | 241 | 1.79 | -0.01 | 0.11 (-0.53, 0.75) | 0.73 | 0.15 (-0.06, 0.35) | 0.16 |
| Any STH | 437 | 3.44 | 0.36 | 241 | 3.78 | 0.47 | 0.11 (-0.39, 0.60) | 0.67 | 0.12 (-0.15, 0.39) | 0.40 |
| **10+** | | | | | | | | | | |
| *A. lumbricoides* | 477 | 2.81 | 0.17 | 250 | 2.53 | 0.11 | -0.10 (-0.60, 0.40) | 0.69 | -0.06 (-0.25, 0.14) | 0.57 |
| *T. trichiura* | 477 | 1.63 | -0.02 | 250 | 2.17 | 0.07 | 0.34 (-0.27, 0.96) | 0.27 | 0.10 (-0.13, 0.32) | 0.41 |
| Any STH | 477 | 4.43 | 0.54 | 250 | 4.69 | 0.59 | 0.07 (-0.36, 0.49) | 0.76 | 0.06 (-0.20, 0.32) | 0.67 |
| **Proportion of children in compound who were dewormed** | | | | | | | | | | |
| **<2/3** | | | | | | | | | | |
| *A. lumbricoides* | 396 | 2.01 | 0.07 | 221 | 2.12 | 0.05 | 0.08 (-0.36, 0.52) | 0.72 | -0.00 (-0.29, 0.28) | 0.97 |
| *T. trichiura* | 396 | 1.32 | -0.10 | 221 | 2.30 | 0.09 | 0.76 (-0.23, 1.75) | 0.13 | 0.21 (-0.14, 0.56) | 0.23 |
| Any STH | 396 | 3.33 | 0.45 | 221 | 4.42 | 0.56 | 0.33 (-0.22, 0.89) | 0.24 | 0.12 (-0.26, 0.50) | 0.53 |
| **2/3+** | | | | | | | | | | |
| *A. lumbricoides* | 516 | 2.58 | 0.09 | 270 | 2.38 | 0.09 | -0.07 (-0.57, 0.42) | 0.77 | 0.00 (-0.22, 0.22) | 0.97 |
| *T. trichiura* | 516 | 1.86 | -0.05 | 270 | 1.72 | -0.01 | -0.07 (-0.47, 0.32) | 0.72 | 0.05 (-0.19, 0.28) | 0.71 |
| Any STH | 516 | 4.45 | 0.46 | 270 | 4.10 | 0.51 | -0.08 (-0.48, 0.33) | 0.71 | 0.05 (-0.25, 0.36) | 0.73 |
| **Proportion of children in cluster who were dewormed** | | | | | | | | | | |
| **<2/3** | | | | | | | | | | |
| *A. lumbricoides* | 339 | 2.49 | 0.06 | 214 | 1.84 | 0.02 | -0.25 (-0.66, 0.16) | 0.22 | -0.04 (-0.27, 0.20) | 0.76 |
| *T. trichiura* | 339 | 1.42 | -0.05 | 214 | 2.00 | 0.05 | 0.40 (-0.06, 0.86) | 0.09 | 0.10 (-0.11, 0.32) | 0.35 |
| Any STH | 339 | 3.90 | 0.46 | 214 | 3.84 | 0.54 | -0.02 (-0.42, 0.38) | 0.91 | 0.07 (-0.21, 0.35) | 0.61 |
| **2/3+** | | | | | | | | | | |
| *A. lumbricoides* | 483 | 2.33 | 0.10 | 263 | 2.51 | 0.10 | 0.08 (-0.63, 0.78) | 0.83 | -0.00 (-0.22, 0.21) | 0.98 |
| *T. trichiura* | 483 | 1.84 | -0.05 | 263 | 1.83 | 0.01 | -0.02 (-0.90, 0.87) | 0.97 | 0.06 (-0.15, 0.27) | 0.57 |
| Any STH | 483 | 4.17 | 0.48 | 263 | 4.34 | 0.52 | 0.03 (-0.72, 0.78) | 0.94 | 0.04 (-0.22, 0.30) | 0.76 |

STH = soil-transmitted helminth; ari = arithmetic; geo = geometric; ECR = egg count reduction; 95% CI = 95% confidence interval

# Table H: Unadjusted egg count reduction for sanitation intervention vs. control arm for larvated soil-transmitted helminth eggs, by subgroups

|  | **Control** | | | **Sanitation** | | | **Arithmetic** | | **Geometric** | |
| --- | --- | --- | --- | --- | --- | --- | --- | --- | --- | --- |
| **Larvated eggs** | **N** | **ari mean** | **geo mean** | **N** | **ari mean** | **geo mean** | **ECR (95% CI)** | **p-value** | **ECR (95% CI)** | **p-value** |
| **Total** | | | | | | | | | | |
| *A. lumbricoides* | 914 | 1.96 | 0.00 | 491 | 1.83 | -0.02 | -0.07 (-0.42, 0.29) | 0.70 | -0.03 (-0.15, 0.10) | 0.68 |
| *T. trichiura* | 914 | 1.24 | -0.19 | 491 | 1.49 | -0.08 | 0.21 (-0.19, 0.61) | 0.31 | 0.11 (-0.01, 0.24) | 0.07 |
| Any STH | 914 | 3.20 | 0.30 | 491 | 3.32 | 0.35 | 0.04 (-0.27, 0.34) | 0.81 | 0.05 (-0.11, 0.21) | 0.54 |
| **Season** | | | | | | | | | | |
| **Dry** | | | | | | | | | | |
| *A. lumbricoides* | 686 | 2.28 | 0.09 | 371 | 1.98 | 0.01 | -0.13 (-0.51, 0.25) | 0.49 | -0.07 (-0.25, 0.10) | 0.41 |
| *T. trichiura* | 686 | 1.50 | -0.08 | 371 | 1.61 | 0.01 | 0.07 (-0.31, 0.45) | 0.71 | 0.09 (-0.11, 0.29) | 0.36 |
| Any STH | 686 | 3.77 | 0.43 | 371 | 3.59 | 0.43 | -0.05 (-0.36, 0.26) | 0.74 | -0.00 (-0.27, 0.26) | 0.98 |
| **Wet** | | | | | | | | | | |
| *A. lumbricoides* | 228 | 1.03 | -0.25 | 120 | 1.39 | -0.14 | 0.36 (-0.40, 1.12) | 0.35 | 0.12 (-0.16, 0.40) | 0.40 |
| *T. trichiura* | 228 | 0.46 | -0.51 | 120 | 1.11 | -0.36 | 1.45 (-0.35, 3.25) | 0.11 | 0.16 (-0.07, 0.38) | 0.17 |
| Any STH | 228 | 1.48 | -0.08 | 120 | 2.50 | 0.11 | 0.70 (-0.34, 1.74) | 0.19 | 0.21 (-0.15, 0.56) | 0.25 |
| **People per compound** | | | | | | | | | | |
| **<10** | | | | | | | | | | |
| *A. lumbricoides* | 437 | 1.54 | -0.06 | 241 | 1.56 | -0.05 | 0.01 (-0.40, 0.42) | 0.95 | 0.01 (-0.18, 0.19) | 0.95 |
| *T. trichiura* | 437 | 1.28 | -0.23 | 241 | 1.38 | -0.12 | 0.09 (-0.58, 0.75) | 0.80 | 0.12 (-0.06, 0.31) | 0.18 |
| Any STH | 437 | 2.82 | 0.26 | 241 | 2.94 | 0.30 | 0.05 (-0.44, 0.53) | 0.85 | 0.05 (-0.18, 0.28) | 0.67 |
| **10+** | | | | | | | | | | |
| *A. lumbricoides* | 477 | 2.35 | 0.06 | 250 | 2.10 | 0.00 | -0.10 (-0.63, 0.42) | 0.69 | -0.05 (-0.23, 0.13) | 0.56 |
| *T. trichiura* | 477 | 1.20 | -0.15 | 250 | 1.59 | -0.05 | 0.34 (-0.26, 0.94) | 0.27 | 0.11 (-0.10, 0.31) | 0.32 |
| Any STH | 477 | 3.55 | 0.35 | 250 | 3.69 | 0.40 | 0.05 (-0.39, 0.48) | 0.84 | 0.05 (-0.20, 0.31) | 0.67 |
| **Proportion of children in compound who were dewormed** | | | | | | | | | | |
| **<2/3** | | | | | | | | | | |
| *A. lumbricoides* | 396 | 1.70 | -0.02 | 221 | 1.62 | -0.05 | -0.04 (-0.43, 0.35) | 0.84 | -0.03 (-0.26, 0.20) | 0.81 |
| *T. trichiura* | 396 | 0.95 | -0.22 | 221 | 1.78 | -0.01 | 0.89 (-0.21, 1.98) | 0.11 | 0.24 (-0.06, 0.55) | 0.12 |
| Any STH | 396 | 2.65 | 0.28 | 221 | 3.40 | 0.36 | 0.29 (-0.26, 0.84) | 0.30 | 0.09 (-0.26, 0.44) | 0.63 |
| **2/3+** | | | | | | | | | | |
| *A. lumbricoides* | 516 | 2.17 | 0.02 | 270 | 2.01 | 0.00 | -0.08 (-0.62, 0.46) | 0.77 | -0.02 (-0.22, 0.17) | 0.80 |
| *T. trichiura* | 516 | 1.46 | -0.16 | 270 | 1.25 | -0.15 | -0.14 (-0.52, 0.24) | 0.47 | 0.02 (-0.19, 0.22) | 0.87 |
| Any STH | 516 | 3.63 | 0.32 | 270 | 3.26 | 0.34 | -0.10 (-0.53, 0.32) | 0.63 | 0.02 (-0.25, 0.29) | 0.87 |
| **Proportion of children in cluster who were dewormed** | | | | | | | | | | |
| **<2/3** | | | | | | | | | | |
| *A. lumbricoides* | 339 | 2.16 | -0.01 | 214 | 1.46 | -0.07 | -0.33 (-0.72, 0.06) | 0.10 | -0.07 (-0.28, 0.14) | 0.53 |
| *T. trichiura* | 339 | 1.05 | -0.19 | 214 | 1.53 | -0.06 | 0.44 (-0.08, 0.96) | 0.09 | 0.13 (-0.07, 0.33) | 0.20 |
| Any STH | 339 | 3.21 | 0.30 | 214 | 2.98 | 0.32 | -0.08 (-0.50, 0.35) | 0.72 | 0.02 (-0.24, 0.28) | 0.88 |
| **2/3+** | | | | | | | | | | |
| *A. lumbricoides* | 483 | 1.92 | 0.02 | 263 | 2.12 | 0.01 | 0.09 (-0.59, 0.77) | 0.79 | -0.01 (-0.21, 0.18) | 0.88 |
| *T. trichiura* | 483 | 1.42 | -0.16 | 263 | 1.38 | -0.11 | -0.04 (-0.85, 0.77) | 0.92 | 0.05 (-0.14, 0.25) | 0.59 |
| Any STH | 483 | 3.35 | 0.33 | 263 | 3.50 | 0.38 | 0.03 (-0.65, 0.72) | 0.92 | 0.04 (-0.20, 0.28) | 0.74 |

STH = soil-transmitted helminth; ari = arithmetic; geo = geometric; ECR = egg count reduction; 95% CI = 95% confidence interval


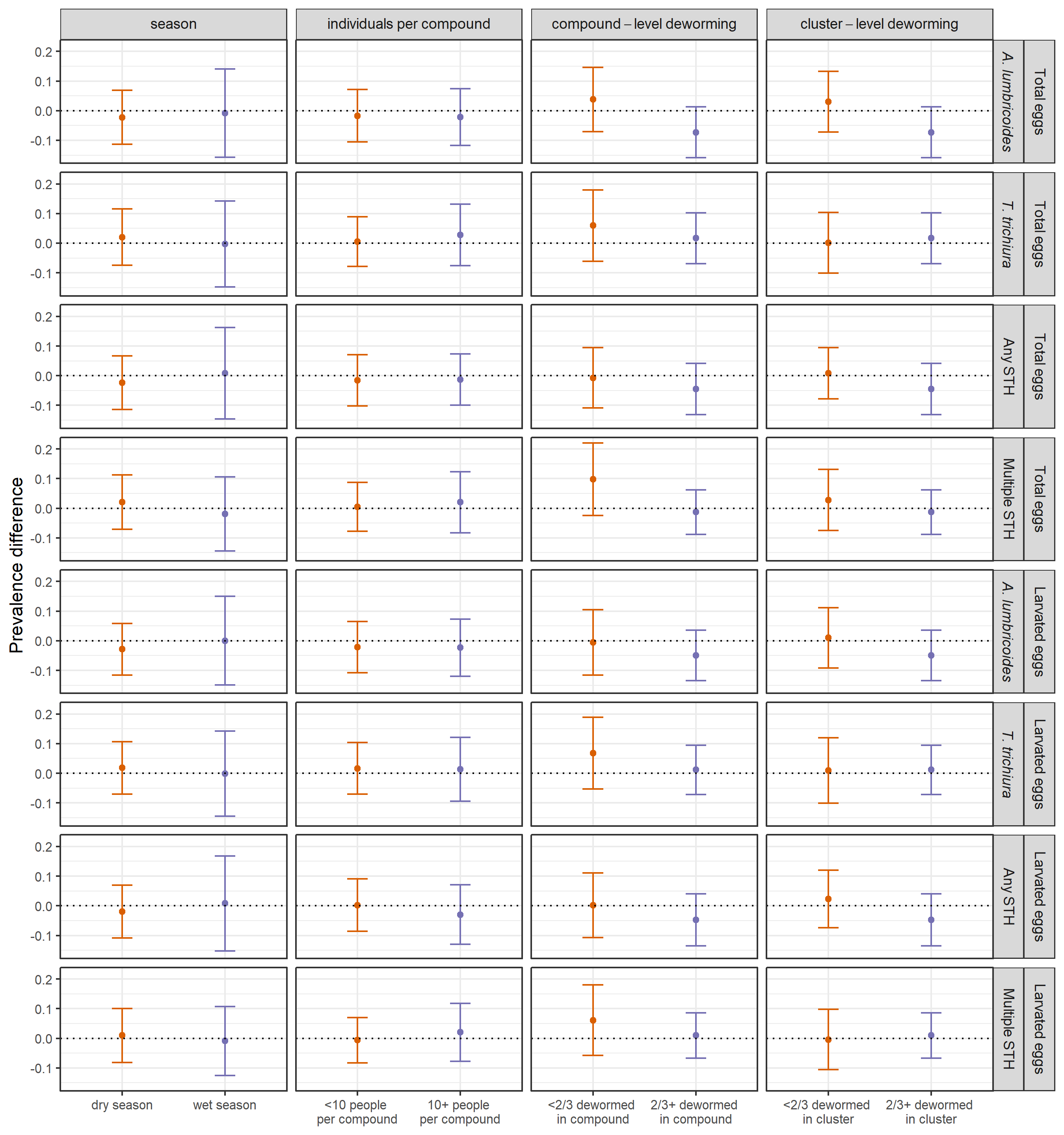


# Fig A: Prevalence difference for total and larvated *A. lumbricoides* eggs, *T. trichiura* eggs, any soil-transmitted helminth eggs, and multiple species of soil-transmitted helminth eggs, by subgroup
