# Supplementary material for: Effect of sanitation improvements on soil-transmitted helminth eggs in courtyard soil from rural Bangladesh: Evidence from a cluster-randomized controlled trial
Source: PLoS Negl Trop Dis. 2021 Jul 28;15(7):e0008815. doi: 10.1371/journal.pntd.0008815 (PMC8351931; doi:10.1371/journal.pntd.0008815)
Supplement: Attachment — Submitted filename: WASHB_STH in soil paper_response to reviewers_210324.docx [file pntd.0008815.s003.docx]

Response to Comments on

“Effect of sanitation improvements on soil-transmitted helminth eggs in courtyard soil from rural Bangladesh: Evidence from a cluster-randomized controlled trial” (EMID:bb51d3b3c9b8c6fb)

*Reviewer #1:*

*1.1 The authors sufficiently describe the study objectives and design. The design is appropriate, high quality, and well-described in this manuscript. Further details on the WASH Benefits study are available elsewhere. The population is clearly described, and the sample size is appropriate. The statistical analysis is appropriate. I have no concerns about ethical issues.*

Thank you for your review of this article and your positive comments.

*1.2: The analysis is appropriate and well described. The results are sufficiently and clearly presented. The figures are clear and appropriate.*

Thank you. We note that we have revised the figures based on comments from Reviewer #2 to further enhance their clarity for readers.

*1.3: The conclusions are supported by the data presented and some limitations are addressed. The authors address the public health relevance of the results.*

Thank you.

*1.4: Line 51: "isolate and hygienically dispose of" - should include "adults"*

We mention containment of adult human feces at the end of the sentence to emphasize that child and animal feces containment need to be in addition to typical adult human feces containment strategies. *1.5: Introduction section - Suggest inclusion of some information about the Bangladesh context; paper generally reflects little if anything about STH epidemiology and STH control in Bangladesh - that's to the detriment of the manuscript in my opinion.*

This is a very helpful suggestion. We have added two sentences to the introduction describing the prevalence of STH infection in Bangladesh and existing MDA coverage:

“The prevalence of STH infection among children aged 2-12 years old in the control arm was 36.8% for *A. lumbricoides,* 9.2% for hookworm, and 7.5% for *T. trichiura* (16)*.* Bangladesh has conducted school-based deworming for school-aged children and immunization-based deworming for pre-school-aged children with mebendazole two times per year since 2008 (33). A 2010 evaluation of the 2009 mass drug administration campaign in two districts of Bangladesh found that for children aged 5-14 years old treatment coverage for one of the twice-annual campaigns was 46-54% depending on district and campaign month (33). Among children aged 4-12 years old enrolled in WASH Benefits, 74% of children in the sanitation arm and 70% of children in the control arm were reported to have received deworming within the last six months (16).”

*1.6: Line 75: Per previous comment - Bangladesh WHO recommends "annual" MDA but Bangladesh treats school-attending school-age children 2x annually, with little documented coverage of non-school attending SAC or preschool age children. Those issues are relevant to your results (including around young children).*

See response to comment 1.5 *1.7: Line 97:  unclear language*

We have revised the language to read “quantifying STH eggs in the soil, a necessary component of the STH life cycle.”  *1.8: Lines 107-9: Suggest moving this sentence to paragraph starting on Line 82*

We decided to keep this sentence in the paragraph that started on line 103 because it reported on STH in environmental reservoirs whereas the studies mentioned in the paragraph that started on line 82 reported on child infection with STH.  *1.9: Lines 123-4: "Eight nearby pregnant..." You mean households with pregnant women? Presumably, you mean nearby to one another.*

We have changed the sentence to “Eight pregnant women in their second or third trimester who lived in nearby households were grouped into clusters and eight adjacent clusters were grouped into a block.” *1.10: Line 143: "structured observations" - observed by who, when?*

The structured observations were conducted by WASH Benefits staff members as part of the intervention uptake assessment; more details are provided in (34). We have revised this sentence to clarify.

“Structured observations of intervention uptake were conducted by WASH Benefits staff members as part of the intervention uptake assessment, approximately 15 months after the intervention delivery and 9 months before our STH assessment.”

*1.11: Line 191: remove "quality assurance." Confirmatory testing would be considered, quality control.*

Thank you for noting that, we have removed “quality assurance”. *1.12: Line 228: "...analyses accounted for [the] lab staff member who..."*

Changed. *1.13: Line 241 & 243: would remove the word, "status" – confusing*

Changed *1.13: Discussion: Discussion is focused and clearly presented. And I would suggest consideration and discussion of the context of the study even though that context is outside of WASHBenefits - it speaks directly to implications and public health relevance. Regarding the comparison with Kenya - the contexts are quite different as you touch on. However, you don't discuss preventive chemotherapy coverage across the entire pop in each country or baseline prevalence.*

We have added a paragraph to the introduction on the STH infection and deworming context of Bangladesh.  *1.14: Lines 335-7: Excellent point and one that I think is central to the broader relevance of the work - but unfortunately not discussed.*

See response to comment 1.16.  *1.15: Line 339-42: You intentionally sampled one small, specific, high-traffic (in front of main entrance to household) area across all HHs, (how) does that affect results?*

We chose to collect soil in front of the household entrance. While it is true that this is a high traffic area, it is also a common area for children who want to be near their caregivers, who are often near of going in and out of the household entrance. Previous work has found similar STH concentrations in soil from in front of the household entrance and in front of the entrance to the latrine. Since every household has a unique household entrance, sampling at the household entrance was a more standardized location. We have modified the description of the location to reflect this.

“Field staff sampled courtyard soil from the area just in front of the entrance of the enrolled household (18). Pervious work has found STH eggs concentration in soil to be similar in front of the household entrance and the latrine entrance (30).”

*1.16: Lines 407-409: See comments from lines 335-7. Suggest that instead of leaving this for the last sentence, a more thorough discussion be had, and evidence (or the lack thereof) from other RCTs/studies of this quality be raised.*

Thank you for this recommendation to expand on the impact of community-level sanitation interventions. We have added the following section to the discussion.

“Community-level, sustained sanitation and/or animal confinement interventions could prevent community-wide STH-contamination of soil, thereby limiting exposure to STH ova and larvae. We are not aware of any studies that have examined the impact of community-wide animal confinement interventions on STH in soil or STH infections in children. With regards to community-level human feces management, previous work has found mixed evidence on whether increased access to latrines is associated with reduced STH infection rates. An observational study in rural Ethiopia found that, after adjusting for individual, household, and community characteristics, hookworm prevalence was not associated with community-level latrine usage (defined as the proportion of households in a cluster that had observed indicators of latrine use such as defined path to latrine and feces observed in the latrine pit) (49). In the same study, *T. trichiura* prevalence and *A. lumbricoides* prevalence were higher in communities where community latrine usage was ≥60% versus <20%. Household latrine usage did not modify the effect of community latrine usage for hookworm or *T. trichiura* prevalence. In communities with latrine usage ≥80%, children in households with a latrine in use had a significantly lower prevalence of *A. lumbricoides* compared to children in households without a latrine. In contrast, in communities with sanitation coverage <20%, children in households with a latrine in use had a significantly *higher* prevalence of *A. lumbricoides* compared to children in households without a latrine. The authors posited that higher community latrine usage may be associated with urbanization, which has been associated with increased STH prevalence and may not have been fully accounted for by including population density in their models. They also suggested that *A. lumbricoides* prevalence in communities with low coverage of latrines may be driven by increased latrine sharing. In India, a cluster-randomized trial of latrine promotion and construction found that 46% of people in the intervention arm had access to a functional latrine at the end of the trial compared to 15% of people in the control arm. Despite this, the intervention had no effect on the prevalence or intensity of hookworm, *A. lumbricoides*, or *T. trichiura* infections (11). In an observational study in Vietnam, 98% of households had latrines, but infection prevalence with STH was 58% for hookworm, 45% for *T. trichiura*, and 14% for *A. lumbricoides* (50). One hypothesis for this finding was that households in this community use fresh night soil as fertilizer. These finding suggest that the impact of community-wide sanitation may be mediated by factors such as household latrine use, latrine sharing, and use of human fecal waste for fertilization, and confounded by factors such as urbanization.”

*1.17: Well-executed study and well-written manuscript. Data analysis is appropriate and well presented. While beyond the immediate aim of WASH Benefits, I would encourage you to more thoroughly discuss the broader relevance of your findings (e.g. need for sustained community-level WASH interventions instead of HH-level interventions, etc.). Obviously, there are practical challenges (e.g. cost) with implementing a similar RCT at community-level.*

See response to comment 1.16.

*Reviewer #2:*

*2.1:  This study is a substudy as part of the larger WASH Benefits clinical trial, and as such the main study population is already defined. The authors clearly state the aim of this paper – to assess the impact of a compound sanitation intervention on the prevalence and abundance of several different species of STH in soil obtained from outside control and intervention households. Overall the results demonstrate that there was no significant impact of the intervention on STH prevalence or abundance. The analyses carried out support the research question outlined, though as noted above, it is difficult to understand how the analysis is conducted with the limited information provided about the R package used (without digging around to find out more yourself). The authors provide a number of interesting and reasonable arguments to support why no significant difference was observed between control and intervention arms for a number of parameters. For example, how it was common to top up soil in courtyards (and so STH could be introduced this way), or how household members could walk STH into the courtyard from other nearby soil on their shoes*

*This paper was interesting, and I believe important findings and ideas are presented that will be very interesting to others working in this area. In general, the details in the methods and results need tightening up, with additional definitions/clarifications added to avoid confusion or misunderstanding by the reader. For example, an explanation for how significance is assessed; acknowledgement of unmeasured confounders (such as household behaviour with regard to sanitation usage and child faeces disposal practices) is also important – is this information available and could it be included in the adjusted analysis?; and more information about how the analysis was carried out (what the R package does). My recommendation would be to revise all of the tables and figures (excluding the sociodemographics table, which is fine) to make them easier to navigate, and a lot more concise, and by moving less important information to the supplementary figure section. I believe this paper would be suitable for publication after a moderate revision. Minor revisions are needed to the text (e.g. additional clarifications), and some more major adjustments to the figures/tables.*

Thank you for reviewing this manuscript.

*2.2: The study design is appropriate, though I would like to see mention of (and ideally inclusion of) additional potential confounders in this study. The authors state that they are primarily interested in unadjusted effects, and state that “as randomisation led to a good balance across arms, bias due to confounding in our dataset is unlikely”. However, the authors mention in the introduction that 94% of adults in the intervention group of the main trial use latrines provided in the intervention, compared with 40% in the control group (though I note it is unclear if this percentage means 40% of control households that already have a hygienic toilet actually use it, or 40% of the whole control population actually have a hygienic toilet). They also report that open defecation is still a problem in both arms, with high percentages of children still practicing OD (e.g. 81% of children aged 0 – 2 years). They also mention that unhygienic disposal of child faeces is also common, with 84% of control and 64% of intervention shouseholds not safely disposing of child faeces. However, none of these factors are measured or adjusted for in the analysis, and I am concerned they may be unmeasured confounders. They seem likely predictors of STH infection of soil in a household compound. Were these factors measured in this subanalysis? If so they need to be adjusted for, and if not, then they need to be mentioned as a limitation or possible explanation for why there is no difference between control/intervention.*

At the baseline measure of the randomized controlled trial, the sanitation and control arm were balanced with respect to hygienic latrine use and safe disposal of child feces which reduces the concern that these factors confound the results.

We modified the paragraph on “intervention uptake” to make it clear that the finding of 94% of households in the sanitation arm and 40% of households in the control arm used a hygienic latrine was from the structured observation conducted *15 months after baseline*. Therefore, these are measurements of intervention output and uptake, rather than inherent baseline differences between the study groups that would confound results. Similarly, the data that 64% of households in the sanitation arm and 84% of household in the control arm practice safe disposal of child feces is also from the same post-intervention structured observation.

“Structured observations of intervention uptake were conducted approximately 15 months after the intervention delivery and 9 months before our STH assessment. At this timepoint, adults were observed to use a hygienic latrine, defined as a latrine with a functional water seal and no feces visible on the latrine slab or floor, in 94% of the all sanitation-arm households and 40% of all control-arm households (34).”

We did not adjust for these factors because we seek to understand the impact of the intervention on STH presence in soil and these factors are indicators of the intervention itself, therefore mediators between the intervention and any environmental impact.

*2.3: The methodology (and introduction) would benefit from a few more definitions. For example, defining what “abundance” means (eggs per gram of soil), and interpreting ICC values e.g. excellent/good/moderate/fair/poor to help the reader. Also, a brief explanation for how prevalence ratios are calculated. They mention “randomisation leading to a good balance”, but would be useful to add “a good balance of sociodemographic characteristics” and add in a reference to table 1…. Again would be good to help the reader by interpreting the ICC values*

We have changed “abundance” to “concentration” throughout the manuscript.

We have added a description of how we defined prevalence and concentration: “Prevalence was defined as the presence of STH eggs of the specified species, and concentration was defined as the number of eggs of the specified species per dry gram of soil.”

We have added a description of the prevalence ratio and prevalence difference to the statistical analysis sub-section of the methods section: “PD was defined as the difference in the prevalence and PR as the ratio of the prevalence in the sanitation vs. control arm.”

The intraclass correlation coefficient is a measure of the variability of STH concentration in different geographic areas (in this case, study blocks). There are no established cut-offs for interpreting intercluster correlation coefficients.

We have clarified that “randomization led to very good baseline balance of sociodemographic and water, sanitation, and hygiene characteristics across arms (Table 1)”.

*2.4: The authors do not provide detail about how the analysis is carried out in R. They mention and reference use of R – “We estimated unadjusted and adjusted intention-to-treat effects using the R (version 3.3.2) package for targeted maximum likelihood estimation with SuperLearner (42,43)”, but no additional information is given. This makes it very difficult for the reader to understand how the analysis was conducted, especially adjusting for potential confounders*

We added a phrase to describe the SuperLearner model: “…SuperLearner is an algorithm that uses cross-validation to test and weight multiple machine learning models to optimize predictive accuracy (43,44).”

*2.5: The data collection also took place over a year (May 2015 – May 2016), which is a long period of time, though I assume this is because of the need to include wet and dry seasons. It would be good to see “time since intervention” included as a measured factor, as I’m not sure how fieldwork was planned/enacted over this long period of time, and if there were different lengths of time between intervention enactment and soil sample analysis (e.g. 12 months for one household, vs, 24 months for others - there could be a significant influence of time between intervention and analysis that could impact the results)*

The intervention was implemented in waves and the soil sample collection followed the same waves so even though data collection took place over 12 months, the time between intervention and soil sample collection was approximately 24 months in all households. The reviewer correctly notes that data collection was over a full year to allow for analysis of differences based on season. We amended the description of the soil collection as follows:

“Soil was collected in a random subset of households enrolled in the sanitation and control arms of the WASH Benefits Bangladesh trial in May 2015-2016. Data collection followed the order of invention roll-out among participating households so that samples were collected approximately 24 months after initiation of interventions in each household. Data collection spanned one year, allowing for comparison of samples collected in the wet and dry seasons.”

*2.6: Authors mention carrying out “replicate” samples, but would be good to state if duplicate or triplicate. The authors mention “Replicates were dropped from the analysis”, but do not say if they were averaged first across replicates, or how they decided which to drop – would be good to explain this.*

Thank you for noting this confusion. We performed replicate analysis in the lab by testing two aliquots from the same sample; the second aliquot was labeled as the replicate. We compared lab replicate results to assess quality and dropped the replicate results before analysis (we did not average the values). We revised the text to additionally include mention of the lab and field blanks, all of which were clean.

“Technical replicates (the second aliquot of a sample analyzed) were dropped from the analysis.”

“There was 0% contamination in blanks.”

*2.7: Authors reference the clinical trial number that this substudy is nested under, but do not mention ethical approval – this should be stated to ensure the study has been approved*

We added this sentence to include the ethical approvals.

“The study protocol was approved by the Ethical Review Committee at The International Centre for Diarrhoeal Disease Research, Bangladesh (PR-11063), the Committee for the Protection of Human Subjects at the University of California, Berkeley (2011-09-3652), and the institutional review board at Stanford University (25863).”

*2.8: The authors should consider if there is a better way to convey the information in the tables and graphs more concisely - they are difficult to navigate as a reader as there is so much information presented*

We appreciate this comment and have condensed the tables and figures in this revision. Please see the response to comment 2.14.

*2.9: I would recommend that the individual graphs in figure 1 are combined into a single graph rather than 8 individual graphs that have the same axes; I would make the same recommendation for figure 2 and 3 – this would also help the reader to compare the different results between species more easily as well as reducing the number of individual graphs.*

Thank you for this suggestion; we have revised the figures to make them easier to read.

*2.10: For tables 2 – 5, I would recommend moving substantial amounts of this data into supplementary figures, and choose only the most important information to include in the main paper. This is a lot of information to include in the main paper, and I don’t feel it adds value to include it all. The authors should decide which are the most important table values to include – some suggestions could be to choose to show “any eggs” results in the main paper and “larvated eggs” results in the supplementary figures? Or just show adjusted figures in the main paper and unadjusted in the supplementary (though I realise the primary parameters chosen are unadjusted so this might not work). Or just show unadjusted overall figures in the main paper and move the sub-analyses results to the supplementary figures. There are a few ways to do it – the authors should decide which are the most important findings to show in the main paper and move the rest to supplementary. It was also difficult to compare adjust vs unadjusted values – adjusted were given as supplementary and unadjusted were in the main tables.*

Our primary outcome was the unadjusted estimates of intervention impact, so we have retained unadjusted results in the main paper. In Table S1 we present both the unadjusted and adjusted results. We also note in the main text that the adjusted and unadjusted analyses yielded similar results. To simplify the presentation, we collapsed the multiple panels of Figure 1 and added the prevalence difference. We also collapse former Figures 2 and 3 into a new Figure 2. Additionally, for readability and ease of interpretation, we moved all of the tables, except Table 1, to the supporting information. We created Figure 3 and Figure 4 to represent the information in the tables and included those in the main manuscript.

*2.11: In the subgroup analyses the authors make a number of statements about differences in STH egg prevalence/intensity values in the dry vs. wet season, and use the word “significant”. The authors need to add a brief explanation/justification as to how they are deciding a finding is significant or not, as there doesn’t seem to be justification/evidence/calculations of how some differences are judged significant and others are not e.g. p values… I am again uncomfortable about mention of “significant differences” without justification in the results section. In the final paragraph of the results section the authors mention modest reductions in prevalence of ascaris in the deworming subgroup analysis – again, it would be good for the authors to expand on how they are determining what is considered a real statistically significant difference and how this is justified - is it just the confidence intervals they are using in this instance?*

We have added a sentences to the methods section to define our use of the term “significant”. We do not use the term “significant” other than in the defined sense of statistically significant.

“We define a result as significant if the corresponding confidence intervals does not cross 1 for prevalence ratios or 0 for differences in counts.”

*2.12: In the opening paragraph of the discussion, the authors mention hygienic latrine use and disposal of child faeces in the intervention arm (“Among households in the sanitation arm, where adults regularly used hygienic latrines but only a third of households safely disposed of child feces the prevalence or abundance of total or larvated A. lumbricoides or T. trichiura eggs in courtyard soil was not significantly lower compared to households in the control arm”). These factors do not appear to have been measured or adjusted for in this substudy analysis. The authors do at least touch on how “continuing child open defecation and unsafe disposal of child feces in the sanitation arm could be a source of STH eggs to contaminate courtyards and contribute to the lack of impact on STH eggs in the environment from the sanitation intervention”, but it would be good to make this clearer and more obvious as a limitation in this study (as it doesn’t seem to have been measured or adjusted for, and would potentially be a strong risk factor).*

These measurements refer to intervention uptake (measured after intervention roll-out) and therefore are a mediator between the interventions and any environmental impact. As such, adjusting for them would erroneously attenuate observed intervention impacts. We view continued open defecation and unsafe feces management as a demonstration that the sanitation intervention was not entirely successful in its user uptake of targeted behaviors. Given the intention-to-treat analysis, the lack of intervention uptake with regard to these behaviors likely contributes to the lack of statistically significant differences in the prevalence and concentration of STH eggs in soil of households in the sanitation arm compared to households in the control arm.

*2.13: The authors mention future use of qPCR to detect STH (especially hookworm, which they weren’t able to detect using their methodology) but I think this could be a stronger recommendation with authors recommending urgent work to validate this method (which is successfully used to detect STH species from faecal samples already) in soil samples.*

Thank you for pointing out that our recommendation could be stronger. We have added the following sentence: “Future work to validate DNA-based methods for detection of STH in soil could improve the reliability of future studies and allow for inclusion of more fragile STH species, such as hookworm.”

*2.14: The main recommendation would be to modify the figures and tables significantly so they are easier to interpret and understand. It may be better for the authors to decide their priorities rather than an editor given there is a lot of data. For example, combine the individual graphs into one for figures 1/2/3, and pick the most important information to present from the tables (rather than presenting everything).*

We have created figures to represent the subgroup analyses and moved the subgroup analysis tables to the supporting information.  *2.15: There are a few minor editorial issues I noticed e.g. “STH eggs in soil have been detected in outdoor soil in various settings” [don’t need to say in soil twice]; “As the method was optimized to detect A. lumbricoides, the recovery efficiency for other STH species was likely was lower” [remove one of the “was” occurrences]; “Based on the daily processing capacity of the lab, we targeted to sample approximately 1500 compounds” [we “aimed/planned/set out to sample” may be better English] etc.*

Thank you for noting these typos; we have made the necessary corrections.

*2.16 [Referring to the abstract]* You have provided percentages for the control households, but it would be useful to see percentages from the intervention households in order to compare.

Since there was no significant difference (no difference for which the confidence intervals did not overlap) between control and intervention households, we chose to present results from control households only. We felt that additionally presenting results from intervention households would give the false impression that they were statistically different.
