# Supplementary material for: Effect of sanitation improvements on soil-transmitted helminth eggs in courtyard soil from rural Bangladesh: Evidence from a cluster-randomized controlled trial
Source: PLoS Negl Trop Dis. 2021 Jul 28;15(7):e0008815. doi: 10.1371/journal.pntd.0008815 (PMC8351931; doi:10.1371/journal.pntd.0008815)
Supplement: Attachment — Submitted filename: WASHB_STH in soil paper_response to editor_210531.docx [file pntd.0008815.s004.docx]

Response to Comments on

“Effect of sanitation improvements on soil-transmitted helminth eggs in courtyard soil from rural Bangladesh: Evidence from a cluster-randomized controlled trial” (EMID:bb51d3b3c9b8c6fb)

*Editor:*

*1.1 Lines 71 & 72: Ancylostoma ceylanicum should now be included in any list of the human hookworms. It is the second most common hookworm of humans in the Asia/Pacific region, with high prevalence in some regions.*

Thank you for noting this; our list now includes *Ancylostoma ceylanicum*.

*1.2 Lines 155-157: What exact month or months of what year were the soil samples examined in this study collected? The degree of rainfall at the time of collection will be very pertinent to results and should be made available to readers.*

On line 180 we previously said that soil samples were collected in May 2015-2016. We revised this to clarify that samples were collected “from May 2015 to April 2016.” We have added the following sentence to describe rainfall in Bangladesh: “Based on data from 1991-2020, the average rainfall in Bangladesh ranges from a low of 7.4 mm in January to 498.0 mm in August.”

*1.3 Lines 197, 202, 208: Please add the manufacturer and city of manufacture for 7X detergent solution, zinc sulfate and sulfuric acid.*

We added the manufacturer of the 7X detergent solution. The zinc sulfate and sulfuric acid we used were generic and therefore we did not add manufacturer information.

*1.4 Lines 227-228. Note also because of the markedly different life cycles of this STH meaning that eggs are less likely to be present in any tested patch of soil [Hookworm larvae hatch after 48-72 hours and seek moisture, so you see them in moisture areas (particularly dewy grass) close to deposited faeces, whereas Trichuris and Ascaris eggs remain viable in the soil for up to three years or more. The hookworm larvae only live for two or three months in the environment. So, you don’t have years worth of hookworm eggs floating around in dust after desiccation of the faeces, to be picked up on hands and shoes and brought into households, as you do with Trichuris and Ascaris].*

We have revised our description of hookworm eggs as follows, “Hookworm eggs, on the other hand, quickly hatch in soil; the larvae seek moisture and can survive in soil for weeks, becoming infective within 2 weeks (19). As such, it is more likely to find *A. lumbricoides* and *T. trichiura* eggs than hookworm eggs in the soil.”

*1.5 Lines 386-402: I assume that household dogs are not present in Bangladesh?  These provide a reservoir of A. ceylanicum hookworm and also disseminate T. trichiura and A. lumbricoides from human faeces via coprophagia. What about cats? They are also a reservoir of A. ceylanicum.*

Thank you asking about the presence of cats and dogs. We added the following sentence to the results, “Few households had animals that can spread STH by consuming human feces (pigs, dogs) and/or are known hosts for the STH species we investigated (pigs, dogs, cats). During all 8 rounds of surveillance, at most 4/750 households (0.5%) owned one or more pigs and 8/750 households (1.1%) owned one or more cats or dogs.” We also modified the discussion to read: “Domestic animals that consume human feces (pig and dogs) or are known hosts for the STH species we investigated (pigs, dogs, and cats) are uncommon in rural Bangladesh. However, the domestic animals that are present may distribute STH eggs across household courtyards.”

*1.6 A. Suum has been shown to be a at most a sub-genotype of A. lumbricoides and more likely the same species, just in a different host.  Pigs may often be infected with the human genotype of A. lumbricoides and vice versa, particularly in LIMC settings. Please change any reference to A. suum to A. lumbricoides. Also note that change line 386, which is incorrect, pigs may be a host for human A. lumbricoides.  You can use the terms “porcine A. lumbricoides” or “porcine ascariasis” if you need to differentiate human from pig infections. Please see links to references supporting this below:*
[*https://academic.oup.com/jid/article/213/8/1355/2459518?login=true*](https://academic.oup.com/jid/article/213/8/1355/2459518?login=true)
[*https://parasitesandvectors.biomedcentral.com/articles/10.1186/1756-3305-5-42*](https://parasitesandvectors.biomedcentral.com/articles/10.1186/1756-3305-5-42)
[*https://www.sciencedirect.com/science/article/pii/S0378111911006494*](https://www.sciencedirect.com/science/article/pii/S0378111911006494)
[*https://www.ncbi.nlm.nih.gov/pmc/articles/PMC4799673/*](https://www.ncbi.nlm.nih.gov/pmc/articles/PMC4799673/)
[*https://bmcvetres.biomedcentral.com/articles/10.1186/1746-6148-10-99*](https://bmcvetres.biomedcentral.com/articles/10.1186/1746-6148-10-99)

Thank you for pointing this out. We modified the discussion to read, “After chickens were fed viable porcine *A. lumbricoides* (formerly known as *A. suum*) (48) and *T. suis* eggs,…” and thereafter refer to *A. suum* as porcine *A. lumbricoides*.
