# Supplementary material for: Effect of sanitation improvements on soil-transmitted helminth eggs in courtyard soil from rural Bangladesh: Evidence from a cluster-randomized controlled trial
Source: PLoS Negl Trop Dis. 2021 Jul 28;15(7):e0008815. doi: 10.1371/journal.pntd.0008815 (PMC8351931; doi:10.1371/journal.pntd.0008815)
Supplement: Attachment — Submitted filename: WASHB_STH in soil paper_response to editor_210619.docx [file pntd.0008815.s005.docx]

Response to Comments on

“Effect of sanitation improvements on soil-transmitted helminth eggs in courtyard soil from rural Bangladesh: Evidence from a cluster-randomized controlled trial” (EMID:bb51d3b3c9b8c6fb)

*Reviewer #3*

*1.1: Lines 294-295:

I think that it is important to clarify which species of STH may cause patent infection in these animals. Currently, these lines read as,
"Few households had animals that can spread STH by consuming human feces (pigs, dogs) and/or are known hosts for the STH species we investigated (pigs, dogs, cats)."

I'd suggest the following potential change,
""Few households had animals that can spread STH by passage after consuming human feces (pigs, dogs) and/or are known hosts for the STH species we investigated (A. lumbricoides in pigs, A. ceylanicum in dogs and cats)."*

Thank you for this clarification. We have made the suggested correction.


*1.2 Lines 392-394:
"Domestic animals that consume human feces (pig and dogs) or are known hosts for Ascaris spp. (pigs, dogs, and cats) are uncommon in rural Bangladesh. However, the domestic animals that are present may distribute STH eggs across household courtyards."

- Cats and dogs are patent hosts for A. ceylanicum, but not hosts for Ascaris (though this is has been suggested for dogs once or twice in the literature, it has not yet been proven). Pigs are true patent hosts for Ascaris.

Suggested alternative-
"Domestic animals are known reservoir hosts for some human-infecting STH (A. lumbricoides in pigs, A. ceylanicum in dogs and cats). These domestic animals are uncommon in rural Bangladesh. However, the domestic animals that are present may distribute STH eggs across household courtyards by passage after coprophagia."*

Thank you for this clarification. We have made the suggested correction.


*1.3 Following these comments being addressed, I have no further revisions.*

We appreciate the reviewer’s sustained effort in reviewing this manuscript to improve its accuracy and clarity.
